# Abnormal liver phosphatidylcholine synthesis revealed in patients with acute respiratory distress syndrome

**DOI:** 10.1194/jlr.P085050

**Published:** 2018-05-04

**Authors:** Ahilanandan Dushianthan, Rebecca Cusack, Michael P. W. Grocott, Anthony D. Postle

**Affiliations:** National Institute for Health Research Southampton Biomedical Research Centre* University Hospital Southampton National Health System Foundation Trust, Southampton SO16 6YD, United Kingdom; Critical Care/Anaesthesia and Perioperative Medicine Research Unit,§ University Hospital Southampton National Health System Foundation Trust, Southampton SO16 6YD, United Kingdom; Integrative Physiology and Critical Illness Group,† Clinical and Experimental Sciences, Sir Henry Wellcome Laboratories, Faculty of Medicine, University of Southampton, Southampton SO16 6YD, United Kingdom

**Keywords:** *methyl*-D_9_-choline, stable isotope, phosphatidylethanolamine *N*-methyltransferase, *S*-adenosyl methionine

## Abstract

Acute respiratory distress syndrome (ARDS) is associated with a severe pro-inflammatory response; although decreased plasma cholesterol concentration has been linked to systemic inflammation, any association of phospholipid metabolic pathways with ARDS has not been characterized. Plasma phosphatidylcholine (PC), the major phospholipid of circulating lipoproteins, is synthesized in human liver by two biologically diverse pathways: the cytidine diphosphocholine (CDP):choline and phosphatidylethanolamine *N*-methyltransferase (PEMT) pathways. Here, we used ESI-MS/MS both to characterize plasma PC compositions and to quantify metabolic fluxes of both pathways using stable isotopes in patients with severe ARDS and in healthy controls. Direct incorporation of *methyl*-D_9_-choline estimated CDP:choline pathway flux, while PEMT flux was determined from incorporations of one and two *methyl*-D_3_ groups derived from *methyl*-D_9_-choline. The results of MS/MS analysis showed significant alterations in plasma PC composition in patients with ARDS versus healthy controls. In particular, the increased overall *methyl*-D_9_-PC enrichment and, most importantly, the much lower *methyl*-D_3_-PC and *methyl*-D_6_-PC enrichments suggest increased flux through the CDP:choline pathway and reduced flux through the PEMT pathway in ARDS. To our knowledge, this study is the first to demonstrate significant plasma PC molecular compositional changes combined with associated alterations in the dynamics of PC synthetic pathways in patients with ARDS.

Acute respiratory distress syndrome (ARDS) is a systemic pro-inflammatory condition associated with significant morbidity and mortality in the intensive care unit (1). Clinically, ARDS is characterized by severe hypoxic respiratory failure at least partly as a consequence of alterations in surfactant phospholipid metabolism leading to alveolar collapse and poor lung compliance (2). However, further questions remain, such as whether these phospholipid changes are only limited to the alveolar environment or whether there are global alterations in phospholipid metabolism; in particular, the hepatic handling of lipids has never been scrutinized before.

Phosphatidylcholine (PC) de novo biosynthesis in humans is dependent on two distinct molecular pathways. While all nucleated mammalian cells are able to synthesize PC by the cytidine diphosphocholine (CDP):choline pathway, hepatocytes can convert phosphatidylethanolamine (PE) into PC by three sequential methylations of PE catalyzed by PE *N*-methyltransferase (PEMT) (3). The preference of PEMT to selectively synthesize PUFA-based PC species highlights the possibility of functional variation between these two synthetic pathways (4). In general, about 30% of hepatic PC is synthesized by PEMT and the reminder by the CDP:choline pathway. As lipid metabolism is highly orchestrated by the liver, alterations in hepatic PC synthesis may have clinical implications in ARDS that have not been previously investigated. Furthermore, PCs are precursors for several biosynthetic pathways of inflammation and resolution (5). Fatty acids esterified to PCs can be liberated by the actions of phospholipase-A_2_ and subsequent generations of oxidative metabolites are regulators of inflammation and have been implicated, at least in part, in the pathogenesis of ARDS (6, 7). In contrast, DHA (*sn*-2 22:6) can also generate mediators that are involved in resolution of inflammation (8). Patients with ARDS characteristically have reduced plasma concentrations of PUFAs (9) and the mechanisms underlying these had not been elucidated. Furthermore, there may be clinical consequences of these alterations in phospholipid handling, influencing the degree of inflammation, initiation of resolution, and progression into chronic inflammation.

PEMT knockout mouse models exhibit a selective reduction in both plasma and hepatic PC species containing DHA (10). We postulate that one possible reason for such reductions in plasma PUFA-PC species in ARDS may be a consequence of alterations in hepatic PEMT-mediated PC synthesis. Traditional lipid quantification techniques using gas chromatography require several multi-step processes, including separation, derivatization, and saponification, which are labor intensive and are not informative regarding molecular species. Stable isotope labeling with *methyl*-D_9_-choline chloride combined with ESI-MS/MS enables assessment of the molecular specificity of PC synthesis via both the PEMT and CDP:choline pathways (4). By quantifying the fractional enrichment of single, double, and triple deuterium-labeled methylated PC species, it is possible to characterize *methyl*-D_3_-*S*-adenosyl methionine (SAMe) enrichment in liver with subsequent estimation of overall PC flux through the PEMT pathway.

## MATERIALS AND METHODS

National ethics committee and University Hospital Southampton research and development approval was obtained (10/WNo01/52 and11/SC/0185). Nonsmokers without any prior medical history were enrolled as controls. ARDS patients were identified according to the American European Consensus Conference diagnostic definition (11). This was based on the following criteria; degree of hypoxemia and bilateral infiltrates on chest radiograph in the absence of clinically raised left atrial pressure. Once assent was obtained, patients were enrolled within 72 h of onset of ARDS. While patients did not receive a controlled diet, they all received enteral feeding (Nutrison™ Nutricia, Trowbridge, UK) through a nasogastric tube within 24 h of admission to the intensive care unit. This provided a goal of achieving 25 kcal/kg body weight per day within 48–72 h and aimed to provide 9.25 mg/kg body weight per day of enteral choline by this time and 5 and 3.4 mg/kg body weight per day, respectively, of the n-3 PUFAs, eicosapentaenoic acid and DHA, by this time. Both patients and controls were infused with 3.6 mg/kg *methyl*-D_9_-choline chloride (Cambridge Isotopes) over 3 h. Blood samples were collected in ethylenediaminetetraacetic acid specimen bottles at 0, 6, 12, 24, 48, 72, and 96 h for patients, and at 0, 8, 24, 48, 72, and 96 h for healthy controls after *methyl*-D_9_-choline chloride infusion. The collected samples were then centrifuged at 400 *g* for 15 min at 20°C. The plasma supernatant was aspirated and stored at −80°C.

### PC analysis

Internal standards (Avanti Polar Lipids) of 10 nmol of dimyristoyl-PC (PC14:0/14:0), 1 nmol of lysophosphatidylcholine (LPC)17:0, and 50 pmol of D_4_-choline were added to 100 μl of plasma. The phospholipid fraction was extracted by chloroform:methanol:water (v/v, 2:2:1) (12). The lower chloroform-rich layer was dried under nitrogen gas at 37°C and was directly infused through MS with an electrospray interface after dissolving with methanol:butanol:water:25% NH_4_OH (6:2:1.6:0.4, v/v) at a rate of 8 μl/min. Collision-induced decomposition resulted in a protonated phosphocholine head group with *m/z* of +184 for PC, *m/z* of +187 for the incorporation of single-deuterated *methyl*-PC group, *m/z* of +190 for double-deuterated *methyl*-PC groups, and *m/z* of +193 for triple-deuterated *methyl-*PC groups. Application of specific precursor ion scans (P184, P187, P190, and P193) resulted in quantification of endogenous and one, two, and three deuterated *methyl*-PC molecular species compositions and concentrations and, hence, enrichment.

### Metabolite analysis

Plasma choline and betaine were analyzed from the upper aqueous phase of the lipid extraction after removal of the lower dichloromethane-rich organic layer. The aqueous phase (2 ml) was freeze-dried and subsequently reconstituted with 100 μl of a 1:1 mixture of acetonitrile and water. Separation was achieved by an Acquity UPLC-Xevo TQ triple quadrupole mass spectrometer system (Waters, Wythenshaw, UK) using a BEH-Hilic column (2.1 × 100 mm × 1.7 μm) with a column temperature of 30°C. The flow rate was 200 μl/min with a starting mobile phase composition of 1% solvent A (ammonium formate 0.5 M/l, pH 4.1 and water) and 99% solvent B (0.5% formic acid in acetonitrile). A linear gradient to 98% solvent A at 4.1 min was followed by re-equilibration to starting conditions at 9 min. MS/MS was used for the mass determination in positive ionization multiple reaction monitoring mode with the following conditions: source temperature 150°C, capillary voltage 3.8 kV, cone voltage 50 V, collision energy 28 eV, desolvation gas (N_2_) flow of 800 l/h at 200°C, collision gas (Ar) flow 0.15 l/min, interscan delay 0.02 s, and dwell time 0.03 s. The labeled and unlabeled fractions of choline and betaine were quantified by multiple reaction monitoring mode using the *m/z* transitions 103.8→59.8 (choline), 112.9→68.9 (D_9_-choline), 117.9→58.9 (betaine), and 126.9→67.9 for D_9_-betaine.

### Plasma cholesterol and triacylglycerol analysis

The plasma lipid profile (HDL-cholesterol, LDL-cholesterol, and triacylglycerol) was quantified by automated Konelab 20 autoanalyzer at NIHR Southampton Biomedical Research Centre. Lipotrol (Thermo Fisher) was used as a quality control sample.

### Data analysis and statistics

The PC ion peaks were quantified using Masslynx software and dedicated in-house Excel spread sheets programmed in Visual Basic. Molecular compositions of major PC species for ion peaks were identified in separate analyses by MS/MS fragmentation of [M-16]^−^ ions in negative ionization and are presented as, for example, PC16:0_20:4, making no assumption about positional distributions of acyl groups (results not shown). Choline and betaine concentrations were calculated by Quanlynx software, corrections for recovery using the D_4_-choline internal standard. The data are presented as mean ± SEM. Comparison of groups was made by unpaired two-tailed Student’s *t*-tests or two-way ANOVA for multiple comparisons using GraphPad Prism (version 5.04).

## RESULTS

### Demographics

Ten healthy volunteers who were nonsmokers without any prior medical problems and ten patients with severe ARDS (PaO_2_/FiO_2_ <100 mmHg) were recruited. The patients were older than the control group (*P* < 0.0001). The choline dose infused per weight was similar between groups (*P* = 0.865). The subjects’ characteristics are listed in **Table 1**.

**TABLE 1.** Patients and control characteristics

	Patients	Controls	*P*
Age	61 ± 5	26 ± 2	<0.0001
Male:female	5:5	6:4	—
Weight (kg)	76 ± 8	78 ± 4	*P* = 0.798
Choline infused (mg)	271 ± 30	277 ± 15	*P* = 0.865

Data are presented as mean ± SEM.

### Plasma lipids, choline, and PC

**Fig. 1.**
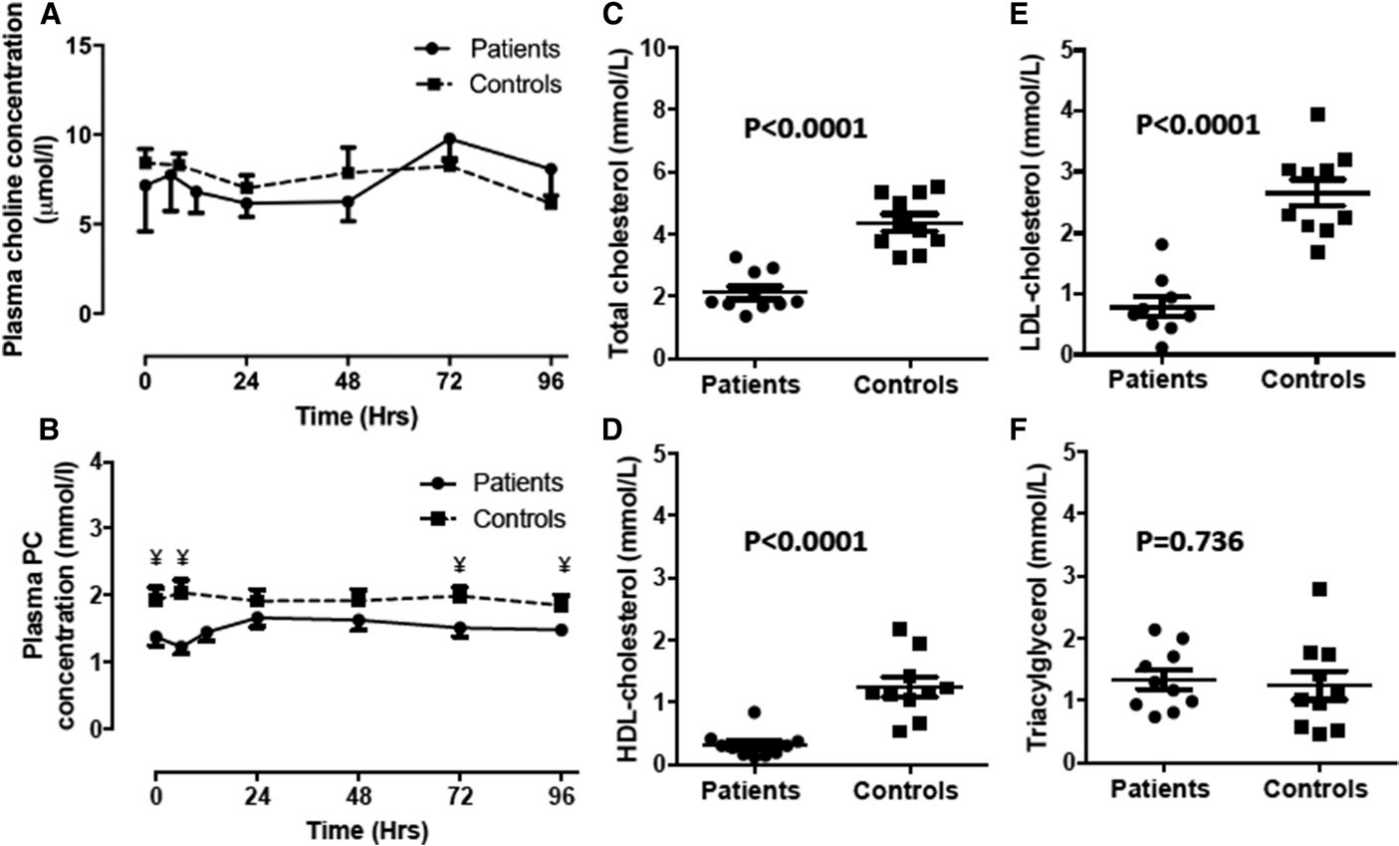
Plasma concentrations (mmol/l) of choline (A), PC (B), total cholesterol (C), HDL-cholesterol (D), LDL-cholesterol (E), and triacylglycerol (F) between ARDS patients and healthy controls. Plasma choline and PC were measured for the duration of the investigative period (4 days). Cholesterol and triacylglycerol concentrations are at time *t* = 0 on day 1. Error bars indicate SEM. Statistical analysis unpaired Student’s *t*-test. ¥, *P* < 0.05.

The plasma choline concentration remained relatively constant over the study period, without any significant difference between patient and control groups. Infusion of *methyl*-D_9_-choline chloride did not alter total choline concentration for either group (**Fig. 1A**). The plasma PC concentration at enrollment tended to be lower in patients (1.45 ± 0.13 mmol/l) than in controls (1.94 ± 0.19 mmol/l) and remained persistently lower throughout the investigative period (**Table 2**), but the difference was only significant at certain time points (0, 6, 72, and 96 h) after *methyl-*D_9_-choline chloride infusion (Fig. 1B). In contrast, there were significant reductions for patient compared with control groups in baseline values of total cholesterol (*P* < 0.0001) (Fig. 1C), HDL-cholesterol (*P* < 0.0001) (Fig. 1D), and LDL-cholesterol levels (*P* < 0.0001) (Fig. 1E). However, triacylglycerol concentrations were similar between both groups (*P* = 0.736) (Fig. 1F).

**TABLE 2.** Fractional plasma PC levels at enrollment

Plasma PC	Patients (mmol/l)	Controls (mmol/l)	Difference
PC16:0_18:2	0.344 ± 0.031	0.593 ± 0.058	Significant
PC16:0_18:1	0.321 ± 0.038	0.276 ± 0.033	Not significant
PC16:0_20:4	0.149 ± 0.018	0.233 ± 0.021	Significant
PC18:1_18:2	0.092 ± 0.008	0.174 ± 0.019	Significant
PC18:0_18:2	0.209 ± 0.026	0.316 ± 0.042	Significant
PC18:0_18:1	0.058 ± 0.010	0.051 ± 0.008	Not significant
PC16:0_22:6	0.050 ± 0.010	0.083 ± 0.007	Significant
PC18:1_20:4	0.038 ± 0.005	0.060 ± 0.006	Significant
PC18:0_20:4	0.095 ± 0.014	0.117 ± 0.013	Not significant
PC18:0_22:6	0.022 ± 0.005	0.024 ± 0.003	Not significant

Data are presented as mean ± SEM. Students *t*-test, *P* < 0.05 is significant.

**Fig. 2.**
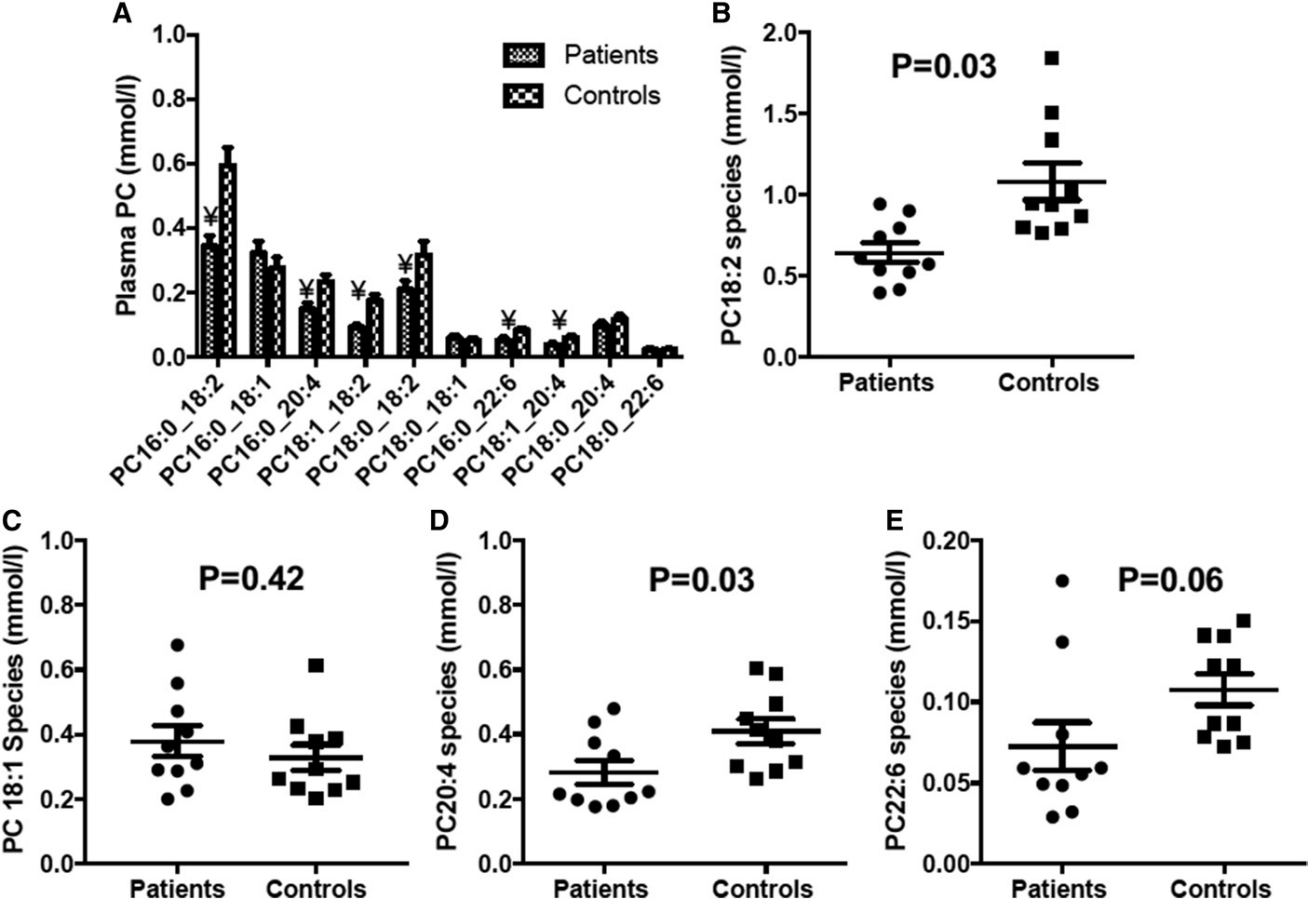
Concentration of selected plasma PC molecular species at enrollment (*t* = 0). A: Mean distribution of PC species. Error bars indicate SEM. Statistical analysis unpaired Student’s *t*-test. ¥, *P* < 0.05. B–E: Scatter plots to illustrate the variation and differences between patient and control groups for selected major (>2% presence) PC species containing 18:2 (B), PC18:1 (C), PC20:4 (D), and PC22:6 (E).

In contrast to the relatively small difference in total plasma PC concentration, ARDS patients exhibited highly significant deficits of selected individual PC species. For instance, plasma concentrations of PC16:0_18:2, PC18:0_18:2, PC18:1_18:2, and PC16:0_20:4 were decreased in this patient group by 42, 34, 47, and 36%, respectively, while concentrations of the monounsaturated species, PC16:0_18:1 and PC18:0_18:1, were unchanged **(****Fig. 2**). Patients had significantly lower concentrations of total di-unsaturated and polyunsaturated PC species with a slight nonsignificant increased concentration of total monounsaturated PC species (Fig. 2B–E).

### *Methyl*-D_9_-choline and *methyl*-D_9_-betaine enrichment

**Fig. 3.**
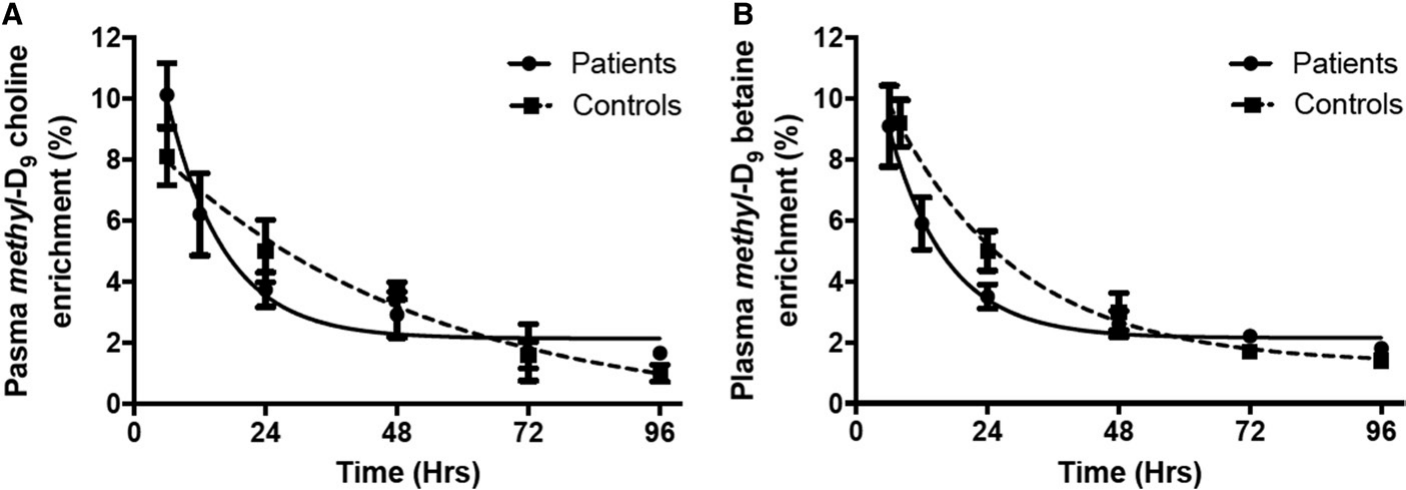
Plasma *methyl-*D_9_-choline (A) and *methyl*-D_9_-betaine (B) enrichments over the total investigative period (4 days). The earliest measured *methyl-*D_9_-choline enrichment, expressed as a percentage of total plasma choline, was 10% and 8% for patients and controls, respectively, with a corresponding value of 9% for *methyl*-D_9_-betaine from both groups. Turnovers of *methyl*-D_9_-choline (*P* < 0.05) enrichment were faster for patient (solid line) compared with control (dashed line) groups (mean ± SEM). A similar trend was observed for *methyl*-D_9_-betaine, although this was not statistically significant. Half-lives were calculated from exponential fits to the data.

Following *methyl*-D_9_-choline infusion, the enrichment of label in plasma choline was similar at the earliest time point for patients (9%) and controls (8%) and was <2% of total plasma choline for both groups by 96 h. Despite these similarities, there was a more rapid decay of labeled choline in patients (*t*1/2 = 7 h) compared with controls (*t*1/2 = 34 h) (*P* < 0.05), indicating an increased turnover of plasma choline, presumably to meet the increased metabolic requirements of the patient group. Choline is oxidized to betaine in the liver and betaine enrichment followed a similar pattern to that of choline. Conversion of choline to betaine was rapid, such that label enrichments were identical at the earliest time point for both groups; there was again a trend for a more rapid decay of *methyl*-D_9_-betaine in patients (*t*1/2 = 7 h vs. 16 h; not significant) and enrichment was <2% after 96 h in both groups (**Fig. 3**).

### *Methyl*-D_9_-, *methyl*-D_3_-, and *methyl*-D_6_-PC enrichment

**Fig. 4.**
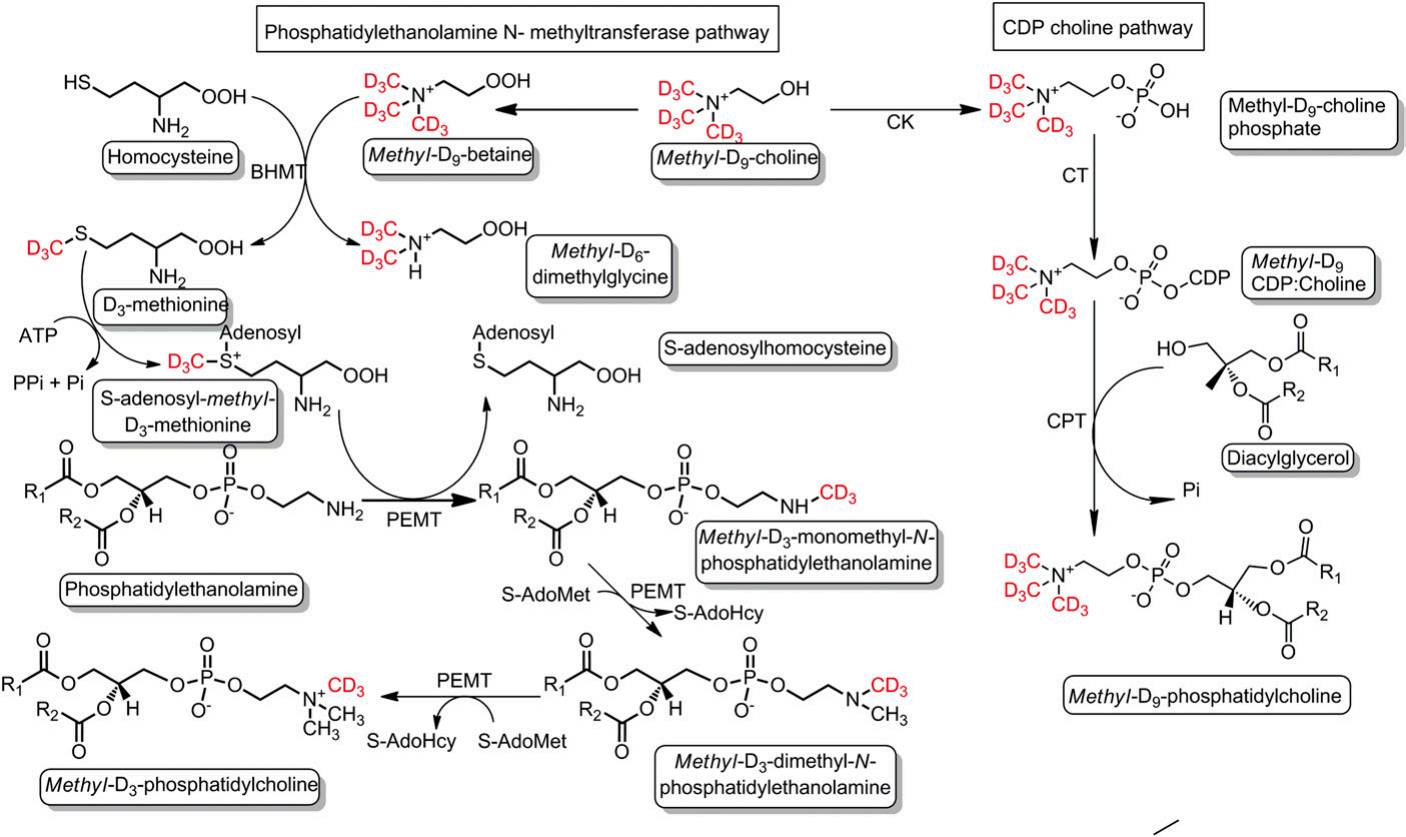
Schematic illustration of PC synthesis and incorporation of labeled *methyl*-D_9_-choline via the CDP:choline and PEMT pathways. Deuterated methyl groups are highlighted as red. The fate of each deuterated methyl group can be traced in individual PC species synthesized by both pathways. CK, choline kinase; CT, phosphocholine cytidylyltransferase; CPT, choline phosphotransferase.

**Fig. 5.**
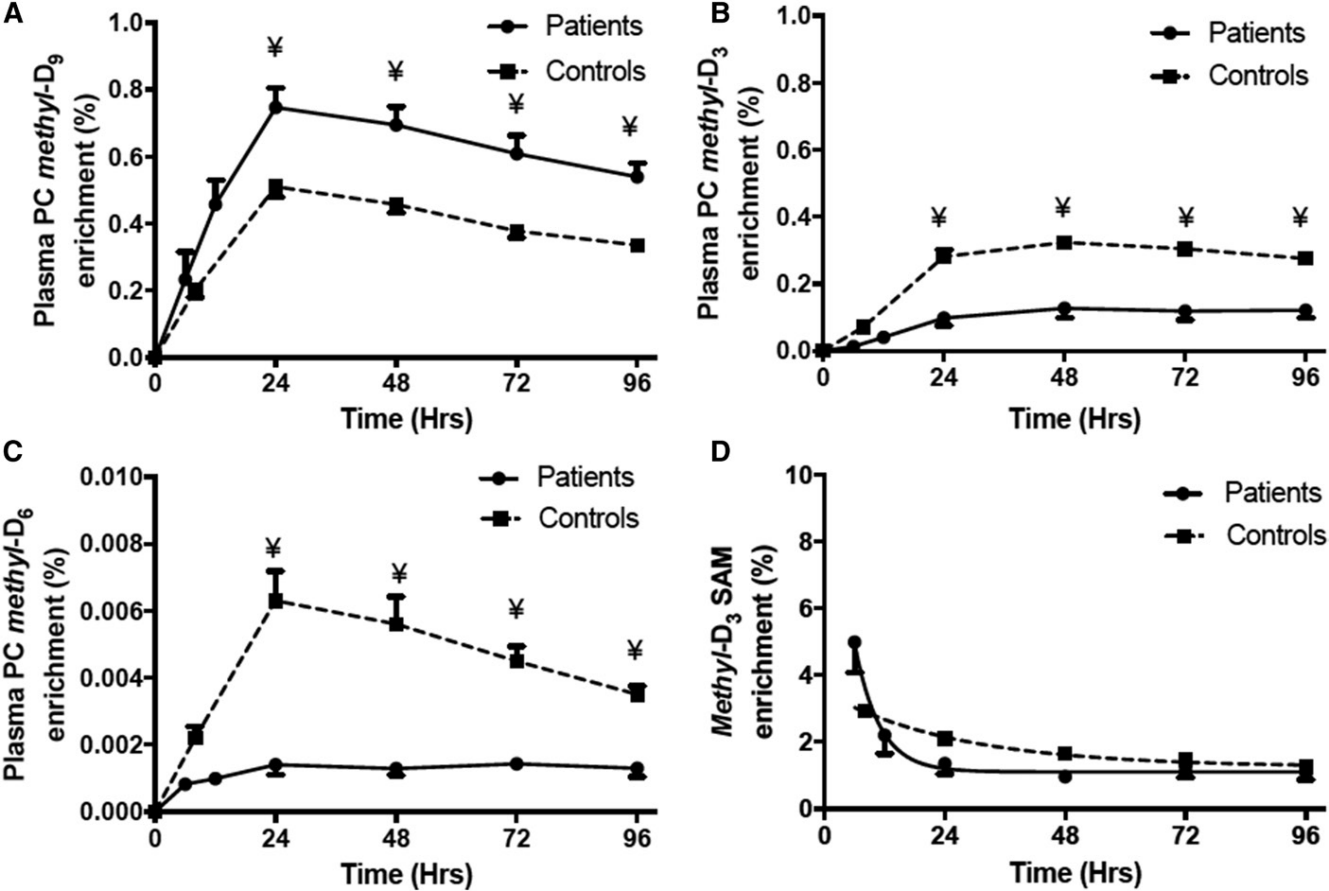
*Methyl-*D_9_-PC (A), *methyl*-D_3_-PC (B), *methyl*-D_6_-PC (C), and *methyl-*D_3_-SAMe (D) enrichment between patients and controls. The PC enrichment was calculated as percentage of total plasma PC pool. *Methyl*-D_3_-SAMe was estimated by P190/Σ(P187 + P190). The solid line represents patients and the dashed line corresponds to controls. Data are expressed as mean ± SEM. ¥, *P* < 0.05. Half-lives for SAMe were calculated from exponential fits to the data and were significantly different (*P* < 0.05) for patient (3.4 h) and control (18.3 h) groups.

Unlabeled PC composition (P184) was monitored by precursor scan of *m/z* 184 (P184) and enrichments of one-*methyl* (D_3_) and two-*methyl* (D_6_) groups, synthesized by the PEMT pathway, and of three-*methyl* groups (D_9_), synthesized by the CDP-choline pathway (**Fig. 4**), were determined by precursor scans of *m/z* 187 (P187), *m/z* 190 (P190), and *m/z* 193 (P193), respectively. There is a theoretical possibility of three *methy*l-D_3_ groups being incorporated into PC by the PEMT pathway, but this would be indistinguishable from direct incorporation of *methyl*-D_9_-choline and would be statistically negligible. In healthy controls, *methyl*-D_9_-PC enrichment was 0.2% of total PC at the earliest time point and subsequently increased to a maximum value at 24 h of 0.51% of total PC. The enrichment pattern of *methyl*-D_9_-PC in the patients’ plasma was identical to that of controls, but with a significantly higher proportional enrichment at and after 24 h (**Fig. 5A**).

In contrast to this increased enrichment of the *methyl*-D_9_-choline in the patients’ plasma PC, enrichments of the *methyl*-D_3_ and *methyl*-D_6_ labels were significantly lower compared with the healthy controls. The maximal enrichment of *methyl*-D_3_-PC was 0.13% of total PC for the patient group compared with 0.32% for the control group (*P* < 0.001) (Fig. 5B). Time courses of label incorporation were comparable for both groups and delayed compared with *methyl*-D_9_ incorporation, being maximal at 48 h. The maximal *methyl-*D_6_-PC enrichment was 0.0014% of total PC for the patient group compared with 0.0063% for the control group (Fig. 5C). The plateauing of *methyl*-D_3_ and -D_6_ enrichment from 24 h is more likely suggestive of breakdown of *methyl*-D_9_-labeled PC than slow *methyl-*D_3_ or -D_6_ incorporation. The lower *methyl-*D_3_ and *methyl*-D_6_ enrichments in patients indicate that the overall flux through the PEMT pathway was significantly reduced compared with healthy controls.

### Quantification of *methyl*-D_3_-SAMe

SAMe is the principle methyl donor for various catalytic reactions, including PC biosynthesis in the liver via the PEMT pathway (Fig. 4). Direct assessment of dynamic biosynthesis of SAMe requires hepatic tissue sampling and is not feasible to study in humans in vivo. For this reason, hepatic *methyl*-D_3_-SAMe enrichment was estimated from the plasma incorporation results using precursor scans of P187 for one methyl group and P190 for two methyl groups from SAMe = P190/Σ(187 + P190). The derivation of this calculation for the quantification of hepatic *methyl*-D_3_-SAMe enrichment has been discussed elsewhere (4). In controls, *methyl*-D_3_-SAMe was maximal at the initial time point (2.92%) and subsequently showed an exponential decay with a half-life of 18.3 h. Although in patients the *methyl*-D_3_-SAMe enrichment was also maximal (4.98%) at the earliest time point, there was a rapid decline with a much shorter half-life of 3.4 h compared with the healthy controls (Fig. 5D; *P* < 0.05). This suggests that the *methyl*-D_3_-SAMe, which is an intermediary for several synthetic pathways, is rapidly metabolized in patients compared with controls.

### Molecular specificity of *methyl*-D_9_-PC, *methyl*-D_3_-PC, and *methyl*-D_6_-PC enrichment

**Fig. 6.**
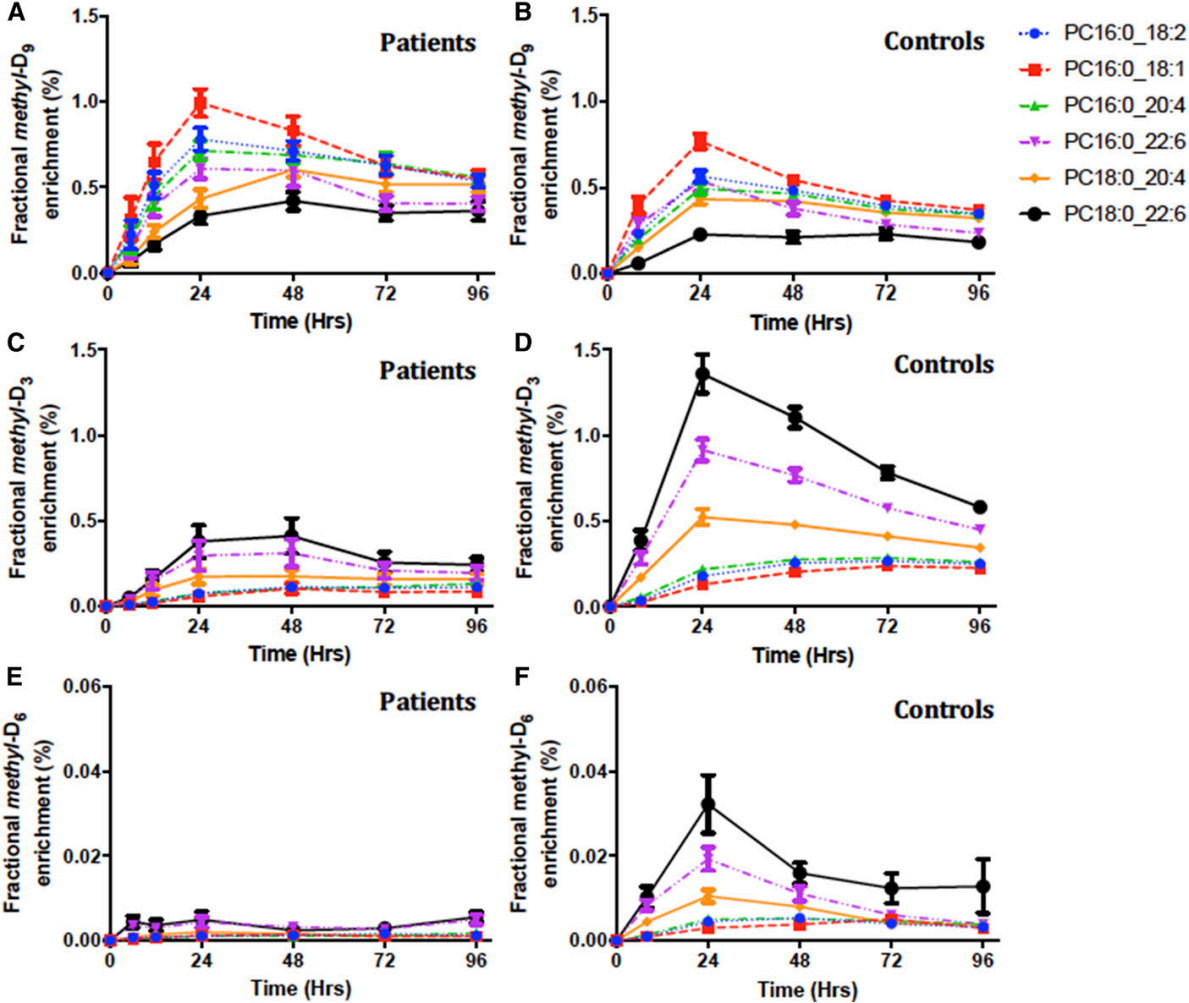
Fractional *methyl*-D_9_-PC (A, B), *methyl*-D_3_-PC (C, D), and *methyl-*D_6_-PC (E, F) enrichment for individual PC species. Results are presented as the labeled fraction in the total plasma PC pool for that individual PC species, compared between patients and control groups over the investigative period for 4 days. Each line color represents individual PC species; blue, PC16:0_18:2; red, PC16:0_18:1; green, PC16:0_20:4; purple, PC16:0_22:6; yellow, PC18:0/20:4; and black, PC18:0/22:6. Data are expressed as mean ± SEM.

The profiles of label incorporation by both the CDP:choline and PEMT pathways masked a considerable variation in terms of individual molecular species and between patients and controls. While the rank order of enrichment of individual species by both pathways was similar for patients and controls, there were significant differences both in maximal enrichments achieved and in the kinetics of label incorporation. The individual species identified in **Fig. 6** were selected as representative of general patterns of incorporation. As reported previously for human volunteers (4), there was preferential incorporation of *methyl-*D_9_-choline into palmitoyl-containing PC species, with maximal enrichments of PC16:0_18:1, for instance, observed at 24 h for controls (0.77 ± 0.04%) and patients (0.99 ± 0.08%) (Fig. 6A, B). By contrast, label incorporations into stearoyl-containing PC species (PC18:0_20:4, PC18:0_22:6) were both lower and delayed, especially for patients for whom these enrichments were maximal at 48 h. This result was consistent with previous studies that reported initial hepatic synthesis of palmitoyl-containing PC species by the CDP:choline pathway, followed by subsequent acyl remodeling into stearoyl-containing species (4).

In direct contrast to the profile of *methyl-*D_9_-choline incorporation, incorporations into both *methyl*-D_3_-PC and *methyl-*D_6_-PC showed very different molecular specificities (Fig. 6C–F). This was most apparent for *methyl-*D_3_ incorporation into a group of PUFA-containing species (PC16:0_22:6, PC18:0_20:4, PC18:0_22:6) with maximal enrichments at 24 h (Fig. 6D), with incorporations into the other three species being lower and maximal at 72 h. We interpret this result as initial incorporation of *methyl-*D_3_ by the PEMT pathway into PUFA-containing PC species, followed by recycling of the labeled choline and its reincorporation into the second groups of species by the CDP:choline pathway. This distinction between pathways was also apparent for patients, although *methyl*-D_3_-PC and *methyl-*D_6_-PC enrichments of the PUFA-containing species were both decreased and delayed for the patient group compared with the control group (Fig. 6C, E).

### Fractional PC synthesis by the PEMT pathway

**Fig. 7.**
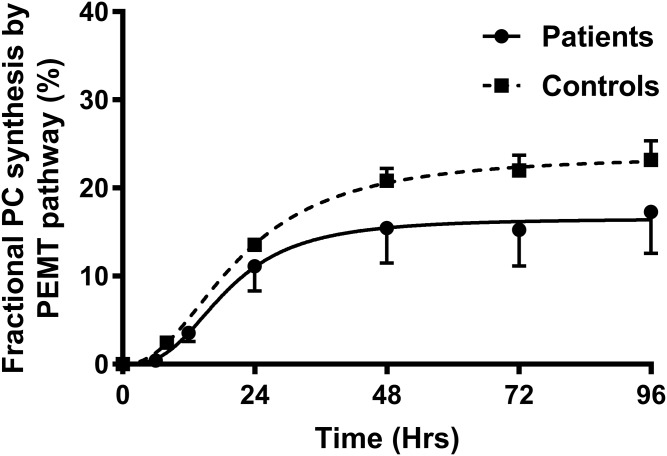
Fractional synthesis of PC via the PEMT pathway. This was estimated by using total *methyl-*D_3_-SAMe corrected for the corresponding *methyl-*D_3_-PC enrichment and presented as the percentage of total PC synthesis. The solid line represents patients and the dashed line corresponds to controls, both calculated as sigmoidal curve fits (*P* < 0.05).

**Fig. 8.**
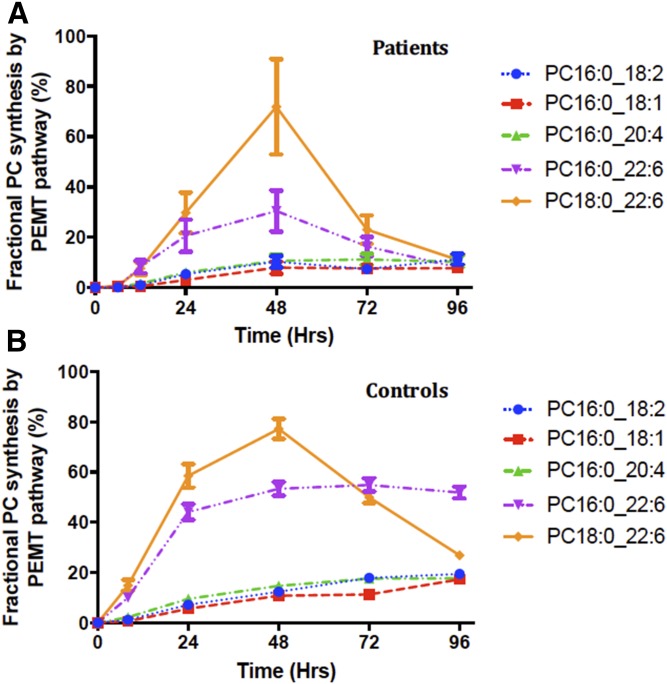
Molecular specificity of PC fractional synthesis via the PEMT pathway. This was estimated by using *methyl-*D_3_-SAMe corrected for the corresponding *methyl-*D_3_-PC enrichment for the particular PC species of interest. Among the selected PC species, PC16:0_22:6 followed by PC16:0_22:6 were the major PC species synthesized by this pathway. Each colored line represents individual molecular PC species: blue, PC16:0_18:2; red, PC16:0_18:1; green, PC16:0_20:4; purple, PC16:0_22:6; and yellow, PC18:0_22:6. Data are expressed as mean ± SEM.

The total PC synthesis through the PEMT pathway was estimated by using values of *methyl*-D_3_-SAMe enrichment corrected for the corresponding *methyl*-D_3_-PC enrichment (**Fig. 7**). In controls, the maximal PEMT-mediated PC synthesis was 23.2 ± 2.2% at 96 h. The total rate of synthesis was 0.3%/h up to 8 h and was maximal at 24 h (0.56%/h). In patients, the PC synthesis through the PEMT pathway was globally reduced for all species. The maximal total PC synthesis via the PEMT pathway was 17.28 ± 4.47% at 96 h. This was an absolute reduction of 25% in PC synthesis by the PEMT pathway in ARDS patients. In controls, the fractional PC synthesis via the PEMT pathway was highest for PC18:0_22:6 (77% of PC species at 48 h) followed by PC16:0_22:6 (53% of PC species at 48 h). Mono- and di-unsaturated PC species showed a slow steady increase in fractional PC synthesis (**Fig. 8**).

### LPC composition and molecular specificity of *methyl*-D_3_-PC and *methyl*-D_6_-PC labeling

**Fig. 9.**
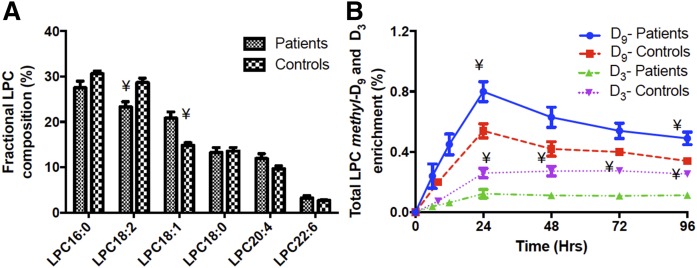
Plasma LPC composition at enrollment (A) and *methyl*-D_9_-LPC and *methyl*-D_3_-LPC enrichments (B) over the investigative period (4 days) for ARDS patients and controls. A significant difference in LPC composition was noted for LPC18:2 and LPC18:1. The LPC enrichment was calculated as percentage of total plasma LPC pool. Blue, *methyl*-D_9_-LPC enrichment for patients; red, *methyl*-D_9_-LPC enrichment for controls; green, *methyl*-D_3_-LPC enrichment for patients; and purple, *methyl*-D_3_-LPC enrichment for controls. Data are expressed as mean ± SEM. ¥*P* < 0.05.

**Fig. 10.**
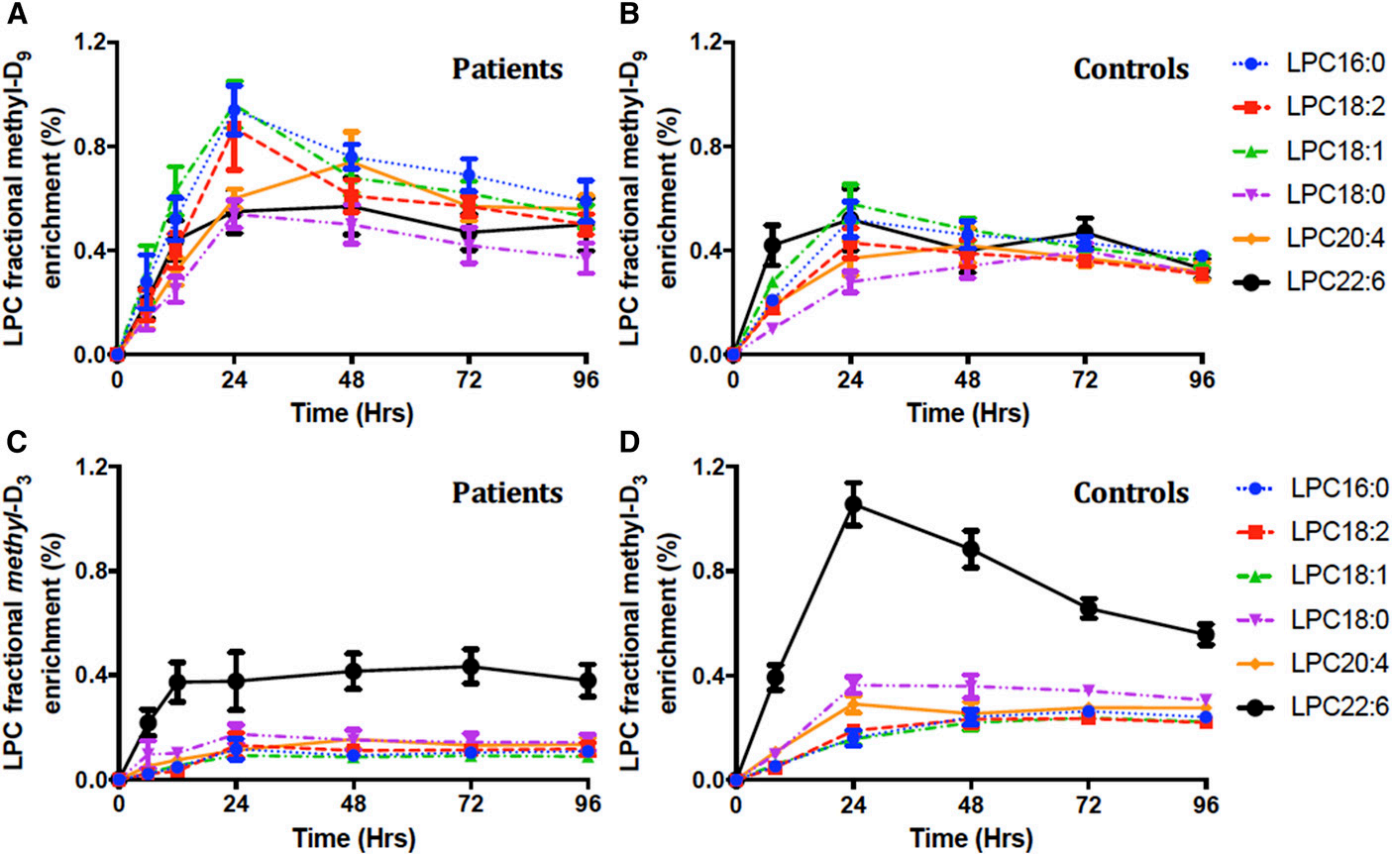
Molecular specificity of fractional *methyl*-D_9_-LPC (A, B) and *methyl*-D_3_-LPC (C, D) enrichment for individual LPC species. Results are presented as the labeled fraction in the total plasma LPC pool for that individual PC species, compared between patient and control groups over the investigative period of 4 days. Each line color represents individual LPC species; blue, LPC16:0; red, LPC18:2; green, LPC18:1; purple, LPC18:0; yellow, LPC20:4; and black, LPC22:6. Data are expressed as mean ± SEM.

The concentration of LPC relative to PC was relatively high in plasma (controls 4.8 ± 0.18%, patients 4.3 ± 0.11%), in contrast to most tissues where LPC amounts are low to minimize its lytic potential. The composition of LPC reflected that of plasma PC, with the fractional concentration of LPC18:1 being increased in patients by 40% and that of LPC18:2 being decreased by 20% (**Fig. 9A**). Our results support animal studies that suggest that circulating LPC is actively secreted from the liver rather than being generated by extrahepatic activity of phospholipase-A_2_ (13). *Methyl*-D_9_ and *methyl*-D_3_ total LPC fractional enrichments (Fig. 9) followed similar temporal patterns to those of plasma PC (Fig. 5A), with comparable maximal values at 24 and 48 h, respectively. Similar to the plasma PC synthesis results, incorporation into total LPC of the *methyl*-D_9_ label was higher and that of the *methyl*-D_3_ label lower in the patient compared with the control group. This result suggests that enrichment of LPC is in equilibrium with that of PC, without the delay that would have been inherent in any significant extra-hepatic hydrolysis. The incorporation patterns of labels into individual molecular species of LPC exhibited a more rapid and greater enrichment of *methyl*-D_9_ in the 16:0, 18:1, and 18:0 species and significantly higher enrichment of *methyl*-D_3_ in LPC22:6 (**Fig. 10**).

## DISCUSSION

ARDS is a multi-organ disease process characterized by a severe form of hypoxic respiratory failure and the ARDS patients in this study were recruited on the basis of their respiratory, not hepatic, symptoms; with only one patient reported with severe liver failure. The decreased concentration of plasma cholesterol in the patient group (Fig. 1) was comparable to previous reports of patients with systemic inflammation and sepsis (14–16), which also showed decreased concentrations of HDL-cholesterol and LDL-cholesterol with maintained concentrations of VLDL and triacylglycerol. The underlying mechanism for this negative temporal association is not clear. In contrast to the cholesterol results, plasma total PC concentration was only marginally decreased throughout the study in the ARDS patient group, suggesting that PC synthesis and secretion was a relatively well-maintained hepatic function in this condition. The lack of change to plasma choline concentration between patient and control groups was expected, as this metabolite is under tight homeostatic control in the relatively short-term, except in response to severe nutritional deprivation (17–19).

This is the first study to demonstrate selective alterations to the composition of plasma PC molecular species in patients with ARDS (Fig. 2A), the details of which suggest nutritional restriction of unsaturated fatty acids. For instance, monounsaturated fatty acids, such as oleate, can be synthesized de novo and, consequently, plasma concentrations of oleate-containing PC species were unchanged in ARDS patients (Fig. 2C). By contrast, n-6 and n-3 fatty acids containing two or more unsaturated double bonds cannot be synthesized de novo in the body and plasma concentrations of virtually all PC species containing these fatty acids were decreased in ARDS patients. The marked decrease in linoleate-containing PC species in ARDS patients (Fig. 2B) is consistent with previous fatty acid analyses of diminished plasma phospholipid linoleic acid concentration in ARDS patients (9). Our results do not support the suggestion in this study that the decrease was due to increased lipid peroxidation, resulting from oxidative stress during systemic inflammation, as we did not observe accumulation of either long- or short-chain PC products of lipid peroxidation in the MS analysis [(20), results not shown]. Additionally, the extent of the decreased concentration was selective depending on the precise molecular species composition, which would not have been expected if this was due solely to peroxidation of plasma PC. For instance, Fig. 2A details greater decreased concentrations of unsaturated PC species containing palmitate at the *sn*-1 position (PC34:2, PC16:0_18:2; PC36:4, PC16:0_20:4; PC38:6, PC16:0_22:6) than of their stearate-containing counterparts (PC36:2, PC18:0_18:2; PC38:4, PC18:0_20;4; PC40:6, PC18:0_22:6). This pattern of change strongly suggests a metabolic origin, as the CDP:choline pathway in the liver preferentially synthesizes *sn*-1 palmitoyl-PC species (21, 22).

In contrast to the unchanged plasma concentration of choline (Fig. 1A), turnover of *methyl*-D_9_-choline (Fig. 3A) and its oxidation product, *methyl*-D_9_-betaine (Fig. 3B), were both significantly increased in ARDS patients, with very similar temporal kinetics. This observation demonstrates the extent of the homeostatic regulation of plasma choline concentration even with increased turnover. As no additional choline was provided in the diet above the recommended daily requirement of 550 mg/day, constant choline concentration in the face of increased turnover implies release of unlabeled choline from tissue stores, probably from hydrolysis of PC in muscle and other tissues. Such a metabolic adaptation to maintain the constant concentration of a metabolite essential for preservation of neuronal activity may be one contributory cause of the rapid and extensive loss of muscle tissue in ARDS patients (23).

The increased fractional enrichment of *methyl*-D_9_-choline incorporation into total plasma PC in ARDS patients (Fig. 5A) further suggests that PC synthesis de novo by the CDP:choline pathway is a preserved pathway in the liver under these conditions of metabolic stress. Increased synthesis and secretion of plasma PC (Fig. 5A) combined with decreased plasma PC concentration (Fig. 1B) possibly indicates minimal disruption of plasma PC secretion in ARDS patients compared with healthy controls. As rates of secretion and catabolism must be balanced at an equilibrium concentration, this suggestion is supported by the lack of increased catabolism and turnover of plasma PC in patients (Fig. 5A). Alternatively, increased uptake of *methyl*-D_9_-choline into the liver in patients, resulting in increased enrichment of label in the hepatic choline metabolite pool supporting PC synthesis, could explain both the rapid turnover of *methyl*-D_9_-choline (Fig. 3A) and the increased enrichment of *methyl*-D_9_-choline PC in plasma from ARDS patients. This possibility could not be addressed in the current study in the absence of liver biopsy. Interestingly, however, we have previously reported enrichment of *methyl*-D_9_-choline label in surfactant PC from lung lavage samples from the same ARDS patients, combined with significantly decreased total PC recovery (2). The similarity of results from two different organs, liver and lungs, where synthesis and secretion of PC is a major function, supports the concept that one mechanism to preserve this critical pathway may be tissue sequestration of plasma choline to support PC synthesis.

In contrast to the CDP:choline pathway, enrichment of plasma PC with the *methyl*-D_3_ (Fig. 5B) and *methyl*-D_6_ (Fig. 5C) demonstrates no comparable preservation of the PEMT pathway in the livers of ARDS patients. PEMT is an intracellular organelle membrane bound enzyme that catalyzes sequential methylations leading to the conversion of PE to PC, and accounts for about 30% of PC production in control hepatocytes. Importantly, it is the only mechanism for synthesis of choline in the body (3), and decreased PEMT activity indicates an increased reliance on dietary choline intake. Such reduced choline synthesis is another potential contributor to the increased plasma choline turnover in ARDS patients (Fig. 3A). The importance of the PEMT pathway has been studied in detail in a variety of mouse models, including the PEMT^−/−^ mouse (10, 24) and mice fed a choline/methionine-deficient diet (25). These animal studies not only highlight the significance of PEMT activity in states of choline deprivation, but also provide supporting evidence for the requirement of this pathway for VLDL secretion and the pathophysiological consequence of steatohepatitis.

To our knowledge, the quantification of *methyl*-D_3_-SAMe enrichment, based on the MIDA analysis of the *methyl*-D_3_- and *methyl*-D_6_-PC enrichments (4), is the first report of increased hepatic SAMe turnover in vivo in a disease state characterized by metabolic dysregulation (Fig. 5D). Whereas previous studies have reported plasma SAMe concentration as a surrogate for its concentration in liver (26), estimation of metabolite flux in liver is a more sensitive indicator of SAMe status. Although direct analysis of liver SAMe concentration was not feasible in the current study, our observation has considerable implications for the whole range of methylation reactions in the body in addition to PEMT activity.

Analysis of the molecular specificity of plasma PC synthesis by the CDP:choline and PEMT pathways in healthy volunteers confirms our previous report (4). *Methyl*-D_9_-choline was incorporated directly initially into *sn*-1 palmitoyl species, followed by a slower incorporation into *sn*-1 stearoyl species (Fig. 6B). Conversely, turnovers of *sn*-1 stearoyl species were considerably slower than those of *sn*-1 palmitoyl species. Plasma PC synthesized by the CDP:choline pathway in ARDS patients followed very similar patterns in terms of individual molecular species (Fig. 2A). Intriguingly, these dynamic changes differ in detail from the compositional analyses presented in Fig. 2, with no enhanced incorporation into PC16:0/18:1 and relatively comparable incorporations into unsaturated PC species. Moreover, the pattern of synthesis of plasma PC species by the PEMT pathway was preserved in ARDS patients (Fig. 2C–F), despite their lower rate of label incorporation (Fig. 5B, C). Incorporation of the *methyl*-D_3_ and *methyl*-D_6_ labels was bi-phasic for both ARDS patients and healthy controls. This incorporation pattern was consistent with an initial synthesis of selected PUFA-containing PC species (PC18:0_22:6, PC16:0_22:6, PC18:0_20:4) by the PEMT pathway, followed by plasma PC recycling back to the liver, release of *methyl*-D_3_-choline and *methyl*-D_6_-choline, and their subsequent delayed incorporation into *sn*-1 palmitoyl species (PC16:0_18:1, PC16:0_18:2, PC16:0_20:4) characteristic of CDP:choline pathway synthesis (Fig. 5A, B). This molecular selectivity for the initial synthesis of polyunsaturated PC species by the PEMT pathway is consistent with the major contributions of PE38:4, PE38:6, PE40:6, and PE36:4 reported for liver PE from healthy volunteers (27). The altered molecular specificity for the CDP:choline pathway in the context of unchanged specificity for the PEMT pathway is probably due to differential compositional modulation of their respective substrate pools of diacylglycerol (DAG). Although all located on the endoplasmic reticulum, the enzymes for the CDP:choline and PEMT pathways are spatially segregated, the former being associated with the Golgi and the latter with the mitochondria (28). We have shown previously, by incubation of isolated rat liver endoplasmic reticulum with either CDP:^14^C-choline or CDP:^14^C-ethanolamine, that compositions of DAG for synthesis of PC and PE are also distinct and differentially regulated (22). Consequently, it is likely that the composition of the DAG substrate pool for CDP:choline phosphotransferase, the last enzyme of the CDP:choline pathway, was altered in the ARDS patients with no change to the composition of the DAG pool that determined the composition of the mitochondrial microsomal PE used for PC synthesis by PEMT. The decreased PEMT flux in the patient group may possibly be due to a decreased ratio of PE to PC, which is reported to regulate PEMT activity in rat liver (29).

The plasma LPC composition generally reflected acyl remodeling mechanisms inherent in hepatic PC synthesis and secretion, containing fatty acids characteristic of both the *sn*-1 and *sn*-2 positions (Fig. 9A), with decreased LPC18:2 and increased LPC18:1 in ARDS patients. The dynamic flux of LPC synthesis (Fig. 9B) was very similar to that of PC (Fig. 2), with enhanced incorporation of *methyl-*D_9_-choline into the plasma PC of ARDS patients and decreased incorporation of *methyl-*D_3_-choline. The suggestion of secretion of 20:4-containing LPC species by rabbit liver for selective transport to peripheral tissues is not supported by our results, which again showed patterns of label incorporation into plasma LPC (Fig. 10) that broadly mirrored the synthetic patterns of the intact PC species in plasma rather than selective enhanced labeling of LPC20:4.

Our findings suggest that patients with ARDS had significantly lower concentrations of total plasma PC, particularly PC16:0_18:2 and PUFA-based species, with a global reduction in total PC flux through the PEMT pathway. This could be due to several reasons. First, ARDS patients tend to have multi-organ involvement, including hepatic dysfunction, and this reduction in synthesis may be an indication of the degree of hepatic synthetic dysfunction endured. However, only one patient in our study had clinically overt liver failure and this patient had dramatically reduced PEMT PC synthesis. Other patients with reduced PEMT PC synthesis had no significant evidence of liver enzyme abnormalities in their plasma. We can hypothesize that this reduction in PEMT activity may reflect early hepatic synthetic dysfunction prior to biochemical evidence of liver damage. Another possibility is the lack of available SAMe for optimal hepatic PC synthesis. The *methy*l-D_3_-SAMe enrichment in patients was higher at the initial time point and showed a rapid decline until 24 h followed by a steady state decay, which was lower than that of controls, indicating that SAMe may be prioritized to maintain methyl balance or used for other methyl-dependent pathways instead of PEMT-mediated hepatic PC synthesis. While no direct evidence is available that increased hepatic SAMe turnover contributes to the pathogenesis of ARDS, increased SAMe-dependent histone methylation is an important component of the inflammatory response in sepsis and cancer (30). Alternatively, enhanced SAMe turnover could have been partly due to decreased synthesis, and inhibition of liver SAMe synthetase has been reported in an endotoxic rat model of septic shock (31).

Studies investigating phospholipid biosynthesis and molecular variations between these synthetic pathways are lacking and are not fully investigated in humans. This is due to the lack of accessibility of human hepatic tissue and methodological limitations in determining in vivo PEMT activity. Because hepatic PC synthesized from PE by PEMT activity is predominantly polyunsaturated, plasma PC-DHA concentration has been proposed as a surrogate for hepatic PEMT activity (32). In our study, however, albeit of limited cohorts of subjects, there was no correlation between concentration of DHA-containing PC species and PEMT flux (results not shown). Stable isotope labeling of choline with deuterium and analytical methods using MS/MS has enabled assessment of hepatic PC molecular synthetic patterns in isolated rat primary hepatocytes and more recently in healthy human volunteers (4), cystic fibrosis patients (33), and pregnant women (34). The study of pregnant women used continuous oral *methyl-*D_9_-choline chloride labeling as opposed to our pulsed infusion. Pulsed infusion of stable isotopes has the advantage of providing information about metabolism of individual molecular species of PC, enabling characterization of the molecular specificity of PEMT activity. In conclusion, this is the first study to apply this dynamic labeling methodology to investigate PC molecular synthetic patterns in a specified disease cohort characterized by systemic inflammatory response and severe hypoxic respiratory failure.
